# The Association of the Levels of High-Density Lipoprotein and Apolipoprotein A1 with SARS-CoV-2 Infection and COVID-19 Severity: An Analysis of the N3C Database

**DOI:** 10.3390/biology12060852

**Published:** 2023-06-14

**Authors:** Meng-Hao Li, Rajendra Kulkarni, Naoru Koizumi, Ali Andalibi, on behalf of the N3C Consortium

**Affiliations:** 1 Schar School of Policy and Government, George Mason University, Arlington, VA 22201, USA; mli11@gmu.edu (M.-H.L.); rkulkarn@gmu.edu (R.K.); nkoizumi@gmu.edu (N.K.); 2 College of Science, George Mason University, Fairfax, VA 22030, USA

**Keywords:** high density lipoprotein, apolipoprotein A1, acute kidney injury, COVID-19, SARS-CoV-2

## Abstract

**Simple Summary:**

This study analyzed data from the National COVID Cohort Collaborative, the largest US COVID-19 database, to determine if an association exists between COVID-19 and blood levels of high-density lipoprotein, and high-density lipoprotein’s main protein component, apolipoprotein A1. Our findings suggest that individuals with higher levels of high-density lipoprotein and apolipoprotein A1 were less likely to be infected with SARS-CoV-2 and were less likely to develop severe COVID-19 requiring hospitalization or invasive medical interventions. They were also less likely to develop acute kidney injury. On the other hand, the presence of underlying health issues (comorbidities) increased the risk of developing severe COVID-19 and acute kidney injury. African American and Hispanic populations were more likely to experience severe disease, while smoking and being male appeared to reduce the incidence of infection but increased the risk of developing severe disease and acute kidney injury. This study is the first to explore the association of high-density lipoprotein and apolipoprotein A1 with COVID-19 outcomes using US population data.

**Abstract:**

This study analyzed data from the National COVID Cohort Collaborative (N3C) database to investigate whether high-density lipoprotein (HDL) and its major protein component, apolipoprotein A1 (apoA1), are associated with severe COVID-19 sequelae, specifically acute kidney injury (AKI) and severe COVID-19 disease as defined by the infection resulting in hospitalization, extracorporeal membrane oxygenation (ECMO), invasive ventilation, or death. Our study included a total of 1,415,302 subjects with HDL values and 3589 subjects with apoA1 values. Higher levels of both HDL and apoA1 were associated with a lower incidence of infection as well as a lower incidence of severe disease. Higher HDL levels were also associated with a lower incidence of developing AKI. Most comorbidities were negatively correlated with SARS-CoV-2 infection, presumably due to the behavioral changes that occurred as a result of the precautions taken by individuals with underlying comorbidities. The presence of comorbidities, however, was associated with developing severe COVID-19 disease and AKI. African American and Hispanic populations experienced worse outcomes, including a higher incidence of infection and the development of severe disease, as well as AKI. Smoking and being male were associated with a lower incidence of infection, while they were risk factors for the development of severe disease and AKI. The results on cholesterol and diabetes drugs warrant further research, given that the database included multiple drugs in each category impeding for analysis of specific medications. Despite the current limitations in the N3C data, this study is the first to investigate the roles of HDL and apoA1 on the outcomes of COVID-19 using the US population data.

## 1. Introduction

Lipoproteins play a very important role in the homeostasis of the body as a whole. High-Density Lipoproteins (HDLs) are around 8 nm in diameter and have a density of 1.063–1.21 g/mL [1]. HDL particles not only transport lipids but are also the carrier of proteins, and other components, thus making HDL an important player in protecting the body against inflammatory assaults [2]. Apolipoprotein A1 (apoA1) plays an important role in lipid metabolism. It is the main component of HDL and helps to remove cholesterol from peripheral tissues to transport back to the liver for disposal [3,4]. Both HDL and apoA1 have been shown to exert anti-inflammatory effects. As such, higher levels of HDL and apoA1 have been shown to be protective against conditions such as cardiovascular disease. In contrast, LDL or low-density lipoprotein, which carries cholesterol from the liver to the rest of the body, does not offer protection and has been shown to be associated with a higher risk of heart disease [5].

Prior studies have shown that both LDL and HDL levels were decreased in patients suffering from coronavirus disease 2019 (COVID-19) caused by the severe acute respiratory syndrome coronavirus 2 (SARS-CoV-2) [6,7]. Later studies demonstrated that higher HDL levels were correlated with a lower risk of SARS-CoV-2 infection [8,9,10], but if infected, could lead to more severe cases of COVID-19, defined as either cases that require hospitalization or cases that result in the death of the patient [6,11,12]. These observations are consistent with the results of studies showing that HDL can protect against LDL oxidation, thus reducing the production of oxidized lipids, such as lipid peroxides and oxidized phospholipids, which are known to be pro-inflammatory [13].

The protective role of HDL in preventing acute kidney injuries (AKI) as a result of COVID-19 is, however, much less explored. Previous studies have shown that higher levels of total cholesterol and LDL are associated with accelerated progression toward end-stage renal disease (ESRD), while low baseline HDL levels in patients with chronic kidney disease (CKD) were linked to higher all-cause mortality [14]. Other studies indicated that lower apoA1 levels are associated with a lower prevalence of CKD [15]. This may be due to the anti-inflammatory and anti-thrombotic effects of HDL, which would reduce atherosclerosis in renal and other arteries [16]. It should be noted that HDL is a complex molecule with structural heterogeneity contributing to functional differences that may not be evident when looking simply at cholesterol-based measurements [17,18].

Given the important role of HDL cholesterol in protecting against inflammation, we analyzed data from the National COVID Cohort Collaborative (N3C) database administered by the National Center of Advancing Translational Sciences (NCATS) under the National Institutes of Health (NIH). The N3C database is currently the largest US-based database that houses a broad range of individual-level data, including demographic, clinical (including results of laboratory tests), patient genomic, and health care service receipt information for the individuals who received COVID-19 tests at clinical settings since 2018. The current study is the first national-level study that leveraged the N3C database to investigate the roles of HDL and apoA1 on outcomes of COVID-19 in the US population. Prior retrospective studies on this topic were conducted either in a single medical center setting [6,7,12], using the UK biobank or the EU population data [8,9,11], or were carried out at a city or state level [10,19]. The current study also compared the roles of HDL and apoA1 in all three aforementioned COVID-19-related outcomes, i.e., SARS-CoV-2 infection, severe cases of COVID-19, which was defined by the need for hospitalization, and the development of AKI as a result of COVID-19, in a single study. The use of the same data to study all three outcomes allowed us to elucidate the roles of HDL and apoA1 in a more comprehensive manner.

## 2. Materials and Method

### 2.1. Data and Data Sources

The access to the N3C database was approved by the investigators’ IRB Review Board on 6 September 2022 under #1936197-2: The study of lipoproteins and their components in SARS-CoV-2 (N3C DUR ID: DUR-44A1395). For the current study, we extracted the data for the period between January 2018 and November 2020. The data after November 2020 were not used to avoid confounding with the COVID-19 vaccination effects.

**Figure 1:**
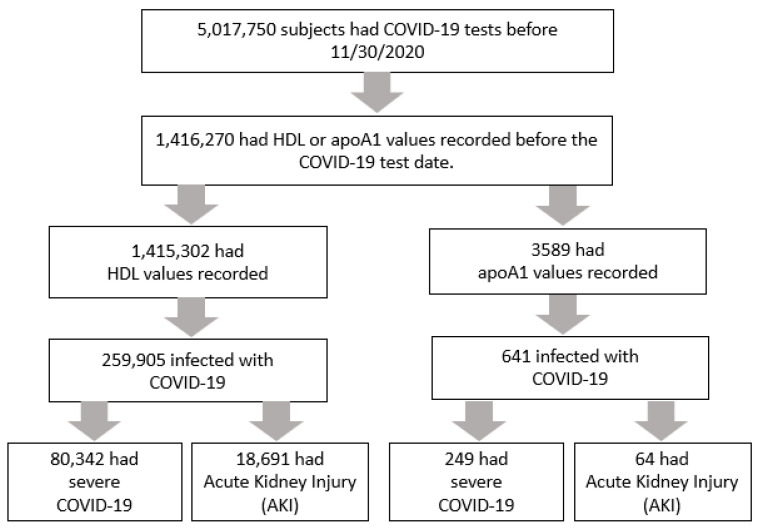
Data extraction flow.

As of 21 March 2023, the N3C database encompassed 18,416,222 subjects. Of those, 5,017,750 received COVID-19 tests before 30 November 2020. Of those, we focused on the subjects whose medical records prior to their COVID-19 tests included either HDL or apoA1 values. There were 1,415,302 subjects with an HDL value and 3589 subjects with an apoA1 value. The subject data were then merged with data on comorbidities and drug use. Of the subjects who had an HDL value recorded, 18.36% (*n* = 259,905) were diagnosed with COVID-19. Of the subjects with an apoA1 value recorded, 17.86% (*n* = 641) were diagnosed with COVID-19. The PRISMA flow diagram summarizing the data extraction process is shown in Figure 1.

### 2.2. Population and Variables

The three outcome variables were considered, including (i) SARS-CoV-2 infection; (ii) severe COVID-19 outcome, i.e., the infection involving hospitalization, and extracorporeal membrane oxygenation (ECMO), invasive ventilation, or death; and (iii) the infection resulting in an acute kidney injury (AKI) requiring dialysis. HDL and apoA1 values recorded prior to the (positive or negative) COVID-19 tests were the two key variables tested for associations with the three outcome measures. The duration between HDL/apoA1 and COVID-19 tests varied by subject. The average duration between HDL and COVID-19 tests was 546 days (SD = 309), while the average duration between apoA1 and COVID-19 tests was 402 days (SD = 318).

A host of demographic, clinical, and other subject-level characteristics were the covariates for the infection as well as the outcomes of COVID-19. Demographic variables included age, sex, and race, while clinical variables were comorbidities known prior to the COVID-19 test, including obesity, diabetes complicated, diabetes not complicated, hypertension, a history of cerebrovascular, heart or lung disease, as well as end-stage kidney disease (ESRD). Other known or suspected risk factors for SARS-CoV-2 infection and outcomes of COVID-19 included smoking, pregnancy, dementia, depression, hemiplegia, HIV, and a history of a transplant. All variables used in this study, along with their corresponding measures, are listed in Appendix A.

### 2.3. Statistical Analysis

Demographic, clinical, and other relevant characteristics were statistically compared between those individuals who: (i) were infected or not infected with SARS-CoV-2; (ii) experienced severe or non-severe COVID-19 as defined above; and (iii) experienced AKI or no AKI as a result of COVID-19. These characteristics were compared in a bivariate fashion using either a *t*-test or Wilcoxon rank-sum test for continuous variables and Chi-sq. or Fisher’s exact test for categorical variables, depending on the sample size and the distribution of the variable tested.

Three multivariable logistic regressions were performed: first, to investigate the risk factors for the SARS-CoV-2 infection, second to investigate the risk factors for the COVID-19 disease severity, and third to investigate the risk factors for developing AKI as a result of COVID-19. The key independent variables in these regressions were HDL and apoA1 values. Since the values of HDL and apoA1 were highly correlated (ρ = 0.70, *p* < 0.001), two separate regressions were run, one with HDL and another with apoA1 values, to address the multicollinearity between the two variables. Running two separate regressions also allowed us to retain more observations in the HDL regression since a large proportion of the subjects only had their HDL values recorded. A total of six logistic regressions were run, two regressions per dependent variable. All statistical analyses were performed using R. Statistical significance was defined by *p* ≤ 0.05 in the analysis unless it is specified otherwise. Following the N3C user agreement, all statistics involving less than 20 observations were presented as “<20” and the associated statistics, such as percentages, were also evaluated at *n* = 20 and were presented accordingly.

## 3. Results

### 3.1. SARS-CoV-2 Infection

Table 1 and Table 2 summarize the results of the bivariate statistical analyses for SARS-CoV-2 infection for samples in which HDL or apoA1 values were measured (HDL group and apoA1 group). Both HDL and apoA1 values were higher in the subjects who tested negative for COVID-19 (54 mg/dL vs. 51 mg/dL with *p* < 0.001 for the HDL group and 144 mg/dL vs. 139 mg/dL with *p* = 0.002 for the apoA1 group). Older age was negatively correlated with the infection (56 y/o vs. 54 y/o, *p* < 0.001) for the HDL group, while it was not significant for the apoA1 group (53.6 y/o vs. 53.0 y/o, *p* = 0.36), the latter result possibly obtained because of the smaller sample size and biased sampling of age groups in the apoA1 group. Sex did not correlate with the risk of infection (57% in both groups, *p* = 0.18) in the HDL group, while females had a higher risk of getting infected in the apoA1 group (46% vs. 52%, *p* of.01) possibly due to a selection bias that may be inherent in the apoaA1 group. Hispanics and Whites were more likely to get infected (4.8% vs. 6.6% for the HDL group, 5.2% vs. 5.5% for the apoA1 group in Hispanics; 69.5% 71.0% for the HDL group, and 66% vs. 71% for the apoA1 group in Whites; all *p* < 0.001 for the HDL group and *p* = 0.002 for the apoA1 group), while Asians were less likely to get infected (3.0% vs. 1.77% for the HDL group, and 4% vs. <3% for the apoA1 group, all *p* < 0.001 for the HDL group and *p* = 0.002 for the apoA1 group). 

Three comorbidities were negatively correlated with the infection in both groups, including obesity (53% vs. 46% for the HDL group and 53% vs. 44% for the apoA1 group; both *p* < 0.001); chronic lung disease (29% vs. 20% for the HDL group, and 35% vs. 27% for the apoA1 group; both *p* < 0.001); HIV (1.2% vs. 0.7% for the HDL group, and 5.9% vs. <3.12% for the apoA1 group; both *p* < 0.001), and depression (29% vs. 21% for the HDL group, and 40% vs. 33% for the apoA1 group; both *p* < 0.001). In addition, complicated diabetes was negatively associated with infection for the HDL group (16% vs. 15%, *p* < 0.001), while uncomplicated diabetes was positively associated with infection in both groups (26.1% vs. 26.5% for the HDL group, and 28% vs. 36% for the apoA1 group; both *p* < 0.001). Most of the other comorbidities, including hypertension (57% vs. 50%), cerebrovascular disease (12% vs. 7%), congestive heart failure (12% vs. 8%), heart failure (15% vs. 11%), hemiplegia (3% vs. 2%), ESRD (2% vs. 1%) and transplant (1% vs. 0.6%), were negatively correlated with infection for the HDL group (*p* < 0.001), but not for the apoA1 group (*p* > 0.05). Smoking was consistently negatively associated with infection (10% vs. 4% for the HDL group and 22% vs. 8% for the apoA1 group; both *p* < 0.001). Pregnancy was negatively associated with infection for the HDL group (4.2% vs. 3.9%, *p* < 0.001), while it was not significant for the apoA1 group (*p* = 0.31). Finally, the use of cholesterol drugs was negatively associated with infection (10% vs. 5% for the HDL group and 11% vs. 4% for the apoA1 group; both *p* < 0.001), and the use of diabetes drugs was positively associated with infection (5% vs. 6% for the HDL group, and 5% vs. 9% for the apoA1 group; both *p* < 0.001).

Table 3 and Table 4 present the results of the multivariable logistic regressions for the infection using HDL and apoA1 groups, respectively. The results were, overall, consistent with the findings of the bivariate analyses. After adjusting for covariates, high HDL reduced the risk of contracting the disease (OR = 0.98, *p* < 0.001). High values of apoA1 were also negatively associated with infection (OR = 0.99, *p* < 0.001). Chronic lung disease (OR = 0.78 with *p* < 0.001 for the HDL group, and OR = 0.76 with *p* = 0.011 for the apoA1 group), obesity (OR = 0.68 for the HDL group, and OR = 0.58 for the apoA1 group; both *p* < 0.001) and HIV (OR = 0.65 for the HDL group, and OR = 0.28 for the apoA1 group; both *p* < 0.001) significantly reduced the risk of infection, while uncomplicated diabetes was a risk factor for infection (OR = 1.24 with *p* < 0.001 for the HDL group, and OR = 1.57 with *p* = 0.001 for the apoA1 group) in both groups.

In the regression, hypertension was the only comorbidity with contradicting results between the two groups. For the HDL group, hypertension reduced the risk of the infection by 9% (OR = 0.91, *p* < 0.001), while it increased it in the apoA1 group (OR = 1.25, *p* = 0.04). Most of the other comorbidities (cardiovascular disease, congestive heart failure, dementia, depression, heart failure, and diabetes complicated) were not statistically significant for the apoA1 group (*p* > 0.05), but they were negatively correlated with the infection for the HDL group (all *p* < 0.001).

Females had a higher risk of infection (OR = 1.21 for the HDL group and OR = 1.39 for the apoA1 group; both *p* < 0.001). Asians had a lower risk of infection than Whites in both groups (OR = 0.45 in the HDL group, and OR = 0.24 for the apoA1 group; both *p* < 0.001) while African Americans (OR = 1.03, *p* < 0.001) and Hispanics (OR = 1.19, *p* < 0.001) had a greater risk of infection in the HDL group. Older age reduced the risk in both groups (OR = 0.997 with *p* < 0.001 for the HDL group, and OR = 0.99 with *p* = 0.04 for the apoA1 group). Pregnancy reduced the infection risk (OR = 0.82, *p* < 0.001) only in the HDL group, while smoking reduced the risk in both groups (OR = 0.39 for the HDL group, and OR = 0.32 for the apoA1 group; both *p* < 0.001). Finally, the use of statins decreased the odds by 51% for the HDL group (OR = 0.49, *p* < 0.001) and by 80% for the apoA1 group (OR = 0.20, *p* < 0.001), while diabetes drugs increased the odds by 35% for the HDL group (OR = 1.35, *p* < 0.001) and by more than 2.5 times for the apoA1 group (OR = 2.55, *p* = 0.001).

### 3.2. SAR-CoV-2 Severity

Table 5 and Table 6 summarize the results of the bivariate statistical analyses for SARS-CoV-2 severity. Both HDL (52 mg/dL vs. 48 mg/dL, *p* < 0.001) and apoA1 (142 mg/dL vs. 135 mg/dL, *p* = 0.01) were negatively associated with the severity. Older age was positively associated with the severity in both groups (51 y/o vs. 61 y/o in the HDL group and 51 y/o vs. 55 y/o for the apoA1 group; both *p* < 0.001). Non-Whites, particularly African Americans, were more likely to have severe disease (11% vs. 19% in the HDL group and 11% vs. 20% for the apoA1 group; both *p* < 0.001). In contrast to the infection case, being female reduced the risk of having a severe disease in both groups (58% vs. 53% with *p* < 0.001 for the HDL group and 55% vs. 47% with *p* = 0.06 for the apoA1 group, which is significant only at the 10% level). In contrast to what was observed for infection, most comorbidities were risk factors for severity in both groups (*p* = 0.03 for obesity for the apoA1 group and *p* < 0.001 for all other statistically significant comorbidities). The only non-significant comorbidities were HIV (*p* = 0.52) and dementia (*p* = 0.14) for the apoA1 group, presumably due to the small sample sizes (both *n* < 20). Pregnancy was negatively correlated with severe disease (4.14% vs. 3.37%, *p* < 0.001) for the HDL group, but it was not statistically significant for the apoA1 group (*p* = 0.16). Conversely, smoking was a risk factor for severe disease for the HDL group (2.66% vs. 5.38%, *p* < 0.001), while it was not statistically significant for the apoA1 group (*p* = 0.210). Finally, the use of both cholesterol (3.27% vs. 8.82%, with *p* < 0.001 for HDL, <5% vs. <8% with *p* = 0.012 for the apoA1 group) and diabetes (4.23% vs. 8.82% with *p* < 0.001 for the HDL group, and 6.38% vs. 12.05% with *p* = 0.012 for the apoA1 group) drugs increased the risk of severe COVID-19.

Table 7 and Table 8 present the results of the multivariable logistic regressions. Both high HDL and apoA1 values reduced the odds of severe COVID-19 (OR = 0.99 with *p* < 0.001 for HDL, and OR = 0.99 with *p* = 0.075 for apoA1 values), although apoA1 was statistically significant only at 10%. Non-Whites were more likely to experience severe disease in the HDL group (ORs ranging between 1.52, Asian, and 2.02, African American, all *p* < 0.001), although African Americans were the only group with a higher risk in the apoA1 group (OR = 1.74, *p* = 0.04). Older age and being male were risk factors (OR = 1.03 and OR = 0.88, respectively; both *p* < 0.001) for the HDL group. For the apoA1 group, both age and sex were statistically non-significant (OR = 1.00 with *p* = 0.59 and OR = 0.81 with *p* = 0.30, respectively). Similarly, both smoking and pregnancy (OR = 1.33 and OR = 1.76, respectively; both *p* < 0.001) were risk factors for the HDL group, while they were not statistically significant for the apoA1 group (OR = 1.00 with *p* = 0.43 and OR = 0.81 with *p* = 0. 80, respectively).

Among the comorbidities, the following variables were risk factors in both groups: cerebrovascular disease (OR = 1.36 with *p* < 0.001 in the HDL group, and OR = 2.12 with *p* = 0.05 for the apoA1 group); diabetes complicated (OR = 1.25 with *p* < 0.001 in the HDL group, and OR = 2.40 with *p* = 0.01 for the apoA1 group); and hemiplegia (OR = 1.78 with *p* < 0.001 for the HDL group, and OR = 9.34 with *p* = 0.01 for the apoA1 group). All other comorbidities were risk factors only for the HDL group (ORs ranging between 1.03, being obese, and 3.13, ESRD, all *p* < 0.001). Finally, the use of cholesterol drugs increased the odds of contracting the severe disease by 31% (OR = 1.31, *p* < 0.001), while the use of diabetes drugs increased the odds of contracting the severe disease by 12% (OR = 1.12, *p* < 0.001) for the HDL group. For the apoA1 group, the use of a cholesterol drug was significant at the 10% level and was found to increase the odds of contracting the severe disease by 137% (OR = 2.37, *p* = 0.08).

### 3.3. Development of AKI

Table 9 and Table 10 summarize the results of the bivariate statistical analysis for the development of AKI as a result of COVID-19. Overall, the findings were similar to those of the severity analysis. Higher HDL and apoA1 values reduced the likelihood of developing AKI (51 mg/dL vs. 46 mg/dL with *p* < 0.001 and 140 mg/dL vs. 135 mg/dL with *p* = 0.085, respectively), although apoA1 values were statistically significant only at the 10% level. Older age was positively associated with AKI in both groups (53 y/o vs. 66 y/o with *p* < 0.001 for the HDL group and 52 y/o vs. 58 y/o with *p* = 0.005 for the apoA1 group). African Americans were more likely to develop AKI (12% vs. 26%, *p* < 0.001) for the HDL group, while females were less likely to develop AKI in both groups (58% vs. 45% with *p* < 0.001 for the HDL group, and 53% vs. 41% with *p* = 0.06 for the apoA1 group) although it was statistically significant only at the 10% level for the apoA1 group. Almost all comorbidities were risk factors for AKI in both groups (*p* = 0.002 for obesity in apoA1 and *p* < 0.001 for all other comorbidities in both groups). The only insignificant comorbidities were HIV for the apoA1 group (*p* = 0.263), most likely due to the small number of HIV patients in the group (*n* < 20). Pregnancy was negatively associated with AKI (4.10% vs. 1.37%, *p* < 0.001) for the HDL group, but it was not statistically significant for the apoA1 group (*p* = 0.10). Smoking was a risk factor for AKI in both groups (3.14% vs. 8.11% for the HDL group and 6.59% vs. 31.25% for the apoA1 group; both *p* < 0.001). The use of cholesterol and diabetes drugs was a risk factor only for the HDL group (4% vs. 12% for cholesterol drugs and 5% vs. 12% for diabetes drugs; both *p* < 0.001).

Table 11 and Table 12 present the results of the multivariable logistic regressions. Higher HDL values reduced the odds of AKI (OR = 0.99, *p* < 0.001), while apoA1 values were not statistically significant (OR = 1.00, *p* = 0.87). Non-whites, regardless of the racial group, were more likely to develop AKI in the HDL group (ORs ranging between 1.34, Hispanic, and 2.05, African American, all *p* < 0.001). However, for the apoA1 group, Hispanics were more likely to develop AKI (OR = 3.13, *p* = 0.05). Older age was a risk factor (OR = 1.03, *p* < 0.001) for the HDL group, while it was not statistically significant for the apoA1 group (OR = 1.00 with *p* = 0.99). Similarly, females were less likely to develop AKI (OR = 0.64, *p* < 0.001), while sex was not statistically significant for the apoA1 group (OR = 0.70, *p* = 0.31). Smoking was a risk factor in both groups (OR = 1.36 with *p* < 0.001 for the HDL group, and OR = 2.31 with *p* = 0.068 for the apoA1 group), although it was statistically significant only at the 10% level for the apoA1 group.

Among the comorbidities, the following variables were statistically significant in both groups: cerebrovascular disease (OR = 1.33 with *p* < 0.001 for the HDL group and OR = 2.35 with *p* = 0.05 for the apoA1 group); and hypertension (OR = 3.35 with *p* < 0.001 for the HDL group, and OR = 3.26 with *p* = 0.02 for the apoA1 group). All other comorbidities were statistically significant only for the HDL group (ORs ranging between 1.14, diabetes uncomplicated, and 2.13, diabetes complicated; both *p* < 0.001). The use of diabetes drugs reduced the risk of AKI by 8% (OR = 0.92, *p* = 0.01) in the HDL group, although it was not statistically significant for the apoA1 group (OR = 1.00, *p* = 0.33). The use of a cholesterol drug was not statistically significant in either group (OR = 1.02 with *p* = 0.46 for the HDL group, and OR = 0.70 with *p* = 0.87 for the apoA1 group).

## 4. Discussion

The current study investigated the relationship of HDL and apoA1 with three different outcomes related to COVID-19, including SARS-CoV-2 infection, the severity of COVID-19, and the development of AKI as a result of COVID-19 in a single study. We investigated these associations using the N3C data, which constitutes the largest COVID-19 database containing data on individuals who were tested for COVID-19 in a clinical setting in the United States. Overall, our results were consistent with those of previous studies, suggesting that higher HDL and apoA1 levels protect against SARS-CoV-2 infection [5,6] as well as severe outcomes of COVID-19 [6,11,12]. More specifically, our results indicated that an increase in the values of HDL and apoA1 reduces the odds of the infection by 2% and 1%, respectively, after adjusting for demographic covariates and comorbidities. Higher values of HDL and apoA1 also reduced the odds of severe COVID-19 by 1%, although the impact of apoA1 was statistically significant only at 0.5 < *p* ≤ 0.1, likely due to the small sample size.

Our study also demonstrated that higher values of HDL were protective against the development of AKI. An increase in HDL levels reduced the odds of developing AKI by 1%, adjusting for covariates. Previous studies have shown that high total cholesterol and LDL levels are associated with accelerated progress toward ESRD, while the anti-thrombotic and anti-inflammatory properties of HDL may protect renal arteries and thus reduce the incidence of kidney damage [14,15]. Furthermore, higher HDL levels were shown to protect patients with sepsis or those following heart and vascular surgery from developing AKI [20]. Our results were consistent with these studies, as well as with in vivo studies of endotoxin-induced renal injury in rats [21,22]. In our study, however, apoA1 was not found to be protective against AKI. While this result may be due to the sample size for the apoA1 group, it may also be attributable to inherent selection bias. Specifically, the patient group in which the apoA1 values were available may, on average, have had more severe cases, and if so, the results could be explained by the fact that patients with severe COVID-19, were treated with steroids, among other drugs. Steroids are known to have a profound effect on immune function as well as on normal physiological processes, including causing an increase in both cholesterol and triglyceride levels, in addition to resulting in sodium and water retention and increase blood pressure [23,24]. Steroid use has been shown to increase LDL and HDL, as well as apoA1 levels in patients with autoimmune disease [25], and short-term steroid use may increase the levels of lecithin cholesterol acetyl transferase (LCAT) and increase levels of HDL2 [26]. As such, the protective effect of apoA1 may simply be muted by the overwhelming stress of SARS-CoV-2 on the body and the potent physiological impact of treatment modalities such as corticosteroids on HDL subpopulations in this group.

It should be noted that HDL is a particle with a significant degree of heterogeneity in size, shape, and composition of associated apoproteins [27]. Moreover, a cholesterol-based test for HDL is unlikely to capture the complexity of the HDL particle and may miss the functional variability that is thought to arise from compositional differences of heterogeneous HDL populations [17,18]. Thus, although total plasma HDL levels are reported as a single number, this number may reflect the presence of varying percentages of the different subfractions in individuals. Additionally, these subfractions are likely to have different biological effects and may be impacted by external factors. 

Moreover, although apoA1 and HDL cholesterol levels seem to have a similar association with the risk of cerebrovascular disease, when the levels of LDL cholesterol and triglycerides are added to the model, the associations can be impacted [27,28]. Such confounding effects have been seen in previous studies. In the Multi-Ethnic Study of Atherosclerosis (MESA) study, HDL particle number was associated with a lower risk of coronary heart disease [29], while the Dallas Heart Study and the Women’s Health Study showed that HDL-cholesterol levels and HDL particle numbers were less correlated [30,31]. The observed differences in the protective effect of HDL in the N3C HDL population and the subpopulation with apoA1 measurements may thus reflect a more nuanced impact that the various subfractions of HDL are having on AKI and COVID-19.

Our study found that the presence of comorbidities, in general, reduced the odds of infection while it increased the odds of severe COVID-19 and AKI. Chronic lung disease and HIV, in particular, reduced the risk of infection by >20% and >30%, respectively. The protective role of these comorbidities most likely reflects the behavioral changes that occurred among the individuals with these conditions as a result of the lockdown. The priming of the immune system by these conditions may also contribute to lower infection rates. In addition, being infected by HIV may confer some protection against other viruses. Interestingly, antibodies that can cross-neutralize SARS-CoV-2 through binding to the heavily glycosylated spike protein have been found in HIV-infected individuals [10]. The only comorbidity found to be a risk factor for infection was uncomplicated diabetes. Complicated diabetes was, on the other hand, a risk factor for getting severe COVID-19 in both groups and for developing AKI in one group. This result may again reflect that the precautionary behavioral changes were more prevalent among those individuals with complicated diabetes. In prior studies, type 2 diabetes mellitus was reported to increase both susceptibility to infection and severity of COVID-19 [22,32,33,34,35]. For COVID-19 severity, chronic lung disease and cerebrovascular disease were found to be risk factors. Both of these are well-established population risk factors for severe COVID-19, as reported in multiple large-scale studies [36,37,38]. For AKI, our risk factors were prior transplant and heart failure. An increased risk of AKI as a result of COVID-19 in transplant recipients is documented, including a meta-analysis that recommends accelerated vaccination programs for kidney transplant recipients [39]. Yet, the effectiveness of the COVID-19 vaccine in this immunocompromised cohort has been debated since the vaccination programs started in November 2020 [40,41,42]. Both cardiovascular disease and heart failure have been associated with AKI in previous studies [43]. As such, the physiological parameters that result in post-vaccination changes may impact some patients adversely, although the greater risks associated with getting COVID-19 are likely to outweigh the side effects of immune hyperactivity that are seen in a small number of vaccine recipients.

Previous studies have shown that males have a higher incidence of COVID-19 and develop more severe disease [44]. In our study as well, males were at a higher risk for severe COVID-19 and AKI. Yet, we saw a higher odds ratio for infections in women. This is inconsistent with the findings of other large studies focused on populations outside the US. Although recent studies have shown that both sexes are equally likely to get infected [44,45], the reason for the discrepancy remains unclear. Our results were consistent with the findings of the prior studies reporting that females are less likely to experience severe COVID-19 [44,46], as well as COVID-19-induced AKI [47]. Older age was weakly associated with a lower incidence of infection in the patient population with HDL values, but it was a risk factor for both severe COVID-19 and AKI. Given the collection dates of the data, it is likely that behavioral factors, such as wearing masks and staying indoors, reduced the incidence of SARS-CoV-2 in older subjects, but that once infected, consistent with other studies, the older patients did have more severe sequelae [19,48]. Similarly, smoking was associated with a lower incidence of infection in both HDL and apoA1 groups though it was a risk factor for both severe COVID-19 and AKI in one group. While this appears to be counterintuitive, nicotine may be playing a mechanistic role, and similar observations have been reported previously, warranting further research on the effects of nicotine [49].

It should be noted, however, that the deleterious effects of smoking outweigh any potential protection that it may confer. Pregnancy was found to be protective against infection in the HDL group while being a risk factor for severe COVID-19 in that population. Prior studies were inconclusive about the effect of pregnancy on the incidence of infection and severity. Studies have suggested that pregnancy increases the risk of contracting COVID-19 due to the weakened immune system [50], while it may or may not increase the odds of developing severe COVID-19 disease. It has also been reported that pregnant women are more likely to be asymptomatic [51], while it has also been reported that pregnancy increases the risk of severe outcomes in COVID-19, especially when infected with the Delta (B.1.617.2) variant of the virus [52]. While it is likely that our null result for the incidence of infection reflects the effect of behavioral changes (greater caution and reduced public exposure) among pregnant women, further research is warranted on the role of pregnancy on the COVID-19 outcomes, especially as the virus continues to evolve. We found minority populations, especially African American and Hispanic populations, are at higher risk for infection, severe COVID-19 and AKI. While there is a general consensus that African American and Hispanic populations had a disproportionately high prevalence, hospitalization rate, and mortality from COVID-19 [29,53], other studies highlighted the differences in COVID-19 severity within ethnic groups. The reasons for these differences, which may be related to immune hyperactivity or other factors, remain to be explained [54].

Finally, our results suggest that using cholesterol-lowering drugs, such as statins and gemfibrozil, reduces the incidence of infection while increasing the odds of developing severe COVID-19. Prior studies have suggested that statins may protect against severe COVID-19 because of their anti-inflammatory and immune-modulatory properties [55,56,57]. However, a study from an Italian hospital reported worse outcomes in COVID-19 patients who are taking statins, calling for caution in accrediting benefits to statin therapy for COVID-19 [58]. The beneficial impact of metformin has also been reported by multiple studies [59,60,61,62,63]. High blood sugar has a profound effect on the immune system, and, as such, it is not surprising that it impacts viral infections, including those with SARS-CoV-2 [64]. Surprisingly, our results suggest that taking diabetes drugs such as metformin increased the risk of both infection and severe COVID-19 while it was protective against AKI, but only in the HDL group. It is currently unclear why there is a discrepancy between our findings and those of others, but it may be that our results are confounded by the underlining comorbidities and thus may not show a statistical benefit from diabetes treatments without further adjustments, such as propensity score matching.

The current study has a few notable limitations. First, although the number of subjects in the N3C database was over 12 million, the sample size was significantly reduced when we restricted the sample to those subjects with complete information on comorbidities as well as data on drug use and lab test results. Moreover, the merging of the data may result in selection bias. HDL heterogeneity may also be a confounding factor. Some of the results, which were inconsistent with prior studies, may at least partially be attributable to the selection bias embedded in the final data or the inherent biological complexity of HDL and its subfractions. Further, N3C data include only those subjects who were tested for COVID-19 in a clinical setting. Thus, those individuals who contracted COVID-19 but did not utilize any healthcare services are automatically excluded. This means that most individuals with very minor and asymptomatic cases of COVID-19 are not captured in the analysis, even though this is true for most, if not all, studies done on this topic. We acknowledge that there is a time lag (up to 1 year) between the time when HDL and apoaA1 values were recorded and the time of COVID-19 testing. Thus, it is possible that HDL and apoaA1 levels changed before the COVID-19 testing. A shorter interval, such as 6 months, may have enhanced the validity of the results, although this would have reduced the sample size significantly, given that a majority of Americans take laboratory tests during their annual physical exams. Lastly, the current study excluded data after November 2020 to avoid the potential confounding effects of COVID-19 vaccinations and subsequent changes in the COVID-19 treatment regimen. As SARS-CoV-2 is constantly mutating to generate new variants, it is possible that the results presented here on the role of HDL and apoA1 may not hold for the newer variants of COVID-19. Continued investigation is needed to better understand the implications of these limitations on our findings.

## 5. Conclusions

The current study demonstrated that higher levels of HDL and apoA1 could reduce the risk of SARS-CoV-2 infection as well as the risks of developing severe cases of COVID-19 and AKI. These associations were established using N3C data, the most comprehensive US-based database that houses a broad range of individual-level data related to COVID-19. While the protective roles of HDL and apoA1 have been well established for the infection and the severity of COVID-19, our study is the first to show the role of HDL in preventing AKI.

Despite the aforementioned caveats, our study validated multiple prior findings in a single study, thereby showing the usefulness of a large population-level database. As SARS-CoV-2 is constantly mutating to generate new variants, it is possible that the results presented here on the role of HDL and apoA1 may not hold for the newer variants of COVID-19. Continued investigation is needed to better understand the implications of these limitations on our findings.

## Figures and Tables

**Table 1:** Descriptive Analysis: SARS-CoV-2 Infection with HDL values.

Variable	Negative (*n* = 1,155,397)	Positive (*n* = 259,905)	*p*-Value ^1^
HDL value (mg/dL), Mean (SD)	53.65 (16.90)	50.71 (15.30)	<0.001
Age, Mean (SD)	56.11 (16.67)	54.28 (16.82)	<0.001
Female, *n* (%)	656,556 (56.83)	147,320 (56.68)	0.184
Race/Ethnicity, *n* (%)			<0.001
White	803,309 (69.53)	184,587 (71.02)	
Asian	34,990 (3.03)	4608 (1.77)	
Black	160,722 (13.91)	34,266 (13.18)	
Hispanic	55,755 (4.83)	17,045 (6.56)	
Other	100,621 (8.71)	19,399 (7.46)	
Pregnancy (yes/no), *n* (%)	48,425 (4.19)	10,147 (3.90)	<0.001
Smoking (yes/no), *n* (%)	112,318 (9.72)	9092 (3.50)	<0.001
Comorbidity (yes/no), *n* (%)			
Obesity	616,601 (53.37)	119,194 (45.86)	<0.001
Hypertension	658,097 (56.96)	129,675 (49.89)	<0.001
Diabetes complicated	190,240 (16.47)	39,747 (15.29)	<0.001
Diabetes not complicated	301,278 (26.08)	68,813 (26.48)	<0.001
Cerebrovascular disease	133,195 (11.53)	18,072 (6.95)	<0.001
Chronic lung disease	332,022 (28.74)	52,513 (20.20)	<0.001
Congestive heart failure	135,533 (11.73)	20,610 (7.93)	<0.001
Heart Failure	177,537 (15.37)	28,613 (11.01)	<0.001
Hemiplegia	33,383 (2.89)	4980 (1.92)	<0.001
HIV	14,306 (1.24)	1899 (0.73)	<0.001
Dementia	46,146 (3.99)	10,264 (3.95)	0.291
Depression	336,067 (29.09)	54,243 (20.87)	<0.001
ESRD	18,926 (1.64)	3705 (1.43)	<0.001
Transplant	11,325 (0.98)	1499 (0.58)	<0.001
Cholesterol drug (yes/no), *n* (%)	114,942 (9.95)	12,953 (4.98)	<0.001
Diabetes drug (yes/no), *n* (%)	57,940 (5.01)	14,678 (5.65)	<0.001

^1^ Statistical tests performed: *t*-, Wilcoxon rank-sum, chi-squared or Fisher’s exact test of independence.

**Table 2:** Descriptive Analysis: SARS-CoV-2 Infection with apoA1 values.

Variable	Negative (*n* = 2948)	Positive (*n* = 641)	*p*-Value ^1^
apoA1 value (mg/dL), Mean (SD)	144.11 (34.29)	139.44 (31.02)	0.002
Age, Mean (SD)	53.56 (14.12)	53.00 (13.72)	0.362
Female, *n* (%)	1350 (45.79)	331 (51.64)	0.007
Race/Ethnicity, *n* (%)			0.002
White	1937 (65.71)	457 (71.29)	
Black	446 (15.13)	93 (14.51)	
Hispanic	153 (5.19)	35 (5.46)	
Asian/Other ^2^	412 (13.96)	56 (8.74)	
Pregnancy (yes/no), *n* (%)	100 (3.39)	27 (4.21)	0.308
Smoking (yes/no), *n* (%)	642 (21.78)	51 (7.96)	<0.001
Comorbidity (yes/no), *n* (%)			
Obesity	1553 (52.68)	284 (44.31)	<0.001
Hypertension	1693 (57.43)	377 (58.81)	0.520
Diabetes complicated	535 (18.15)	134 (20.90)	0.104
Diabetes not complicated	831 (28.19)	228 (35.57)	<0.001
Cerebrovascular disease	291 (9.87)	51 (7.96)	0.135
Chronic lung disease	1042 (35.35)	172 (26.83)	<0.001
Congestive heart failure	336 (11.40)	67 (10.45)	0.492
Heart Failure	468 (15.88)	96 (14.98)	0.571
Hemiplegia	70 (2.37)	<20 (<3.12)	0.440
HIV	172 (5.83)	<20 (<3.12)	<0.001
Dementia	79 (2.68)	<20 (<3.12)	0.856
Depression	1175 (39.86)	213 (33.23)	0.002
ESRD	67 (2.27)	22 (3.43)	0.087
Transplant	40 (1.36)	<20 (<3.12)	0.322
Cholesterol drug (yes/no), *n* (%)	323 (10.96)	26 (4.06)	<0.001
Diabetes drug (yes/no), *n* (%)	149 (5.05)	55 (8.58)	<0.001

^1^ Statistical tests performed: *t*-, Wilcoxon rank-sum, chi-squared or Fisher’s exact test of independence. ^2^ According to the N3C DUA, Asian (*n* < 20; <3.12%) was combined with Other to increase the cell size and avoid back-calculation. The statistical test was performed on the data before combining the two categories.

**Table 3:** Logistic Regression for SARS-CoV-2 Infection with HDL as a Key Variable (*n* = 1,415,302).

Variable	OR ^1^	95% CI ^1^	*p*-Value
HDL value (mg/dL)	0.98	0.98, 0.98	<0.001
ESRD	1.20	1.15, 1.25	<0.001
Obesity	0.68	0.67, 0.69	<0.001
Pregnancy	0.82	0.80, 0.84	<0.001
Transplant	0.63	0.60, 0.67	<0.001
Smoker	0.39	0.38, 0.40	<0.001
Cerebrovascular disease	0.68	0.67, 0.70	<0.001
Chronic lung disease	0.78	0.77, 0.79	<0.001
Congestive heart failure	0.87	0.85, 0.90	<0.001
Dementia	1.31	1.28, 1.34	<0.001
Depression	0.72	0.71, 0.73	<0.001
Diabetes complicated	0.97	0.95, 0.99	<0.001
Diabetes not complicated	1.24	1.22, 1.26	<0.001
Heart Failure	0.97	0.95, 1.0	0.018
Hemiplegia	1.01	0.98, 1.05	0.462
HIV	0.65	0.61, 0.68	<0.001
Hypertension	0.91	0.90, 0.92	<0.001
Cholesterol drug	0.49	0.48, 0.50	<0.001
Diabetes drug	1.35	1.33, 1.38	<0.001
Age	0.997	1.00, 1.00	<0.001
Female	1.21	1.20, 1.22	<0.001
Race ^2^			
Black	1.03	1.01, 1.04	<0.001
Asian	0.45	0.44, 0.46	<0.001
Hispanic	1.19	1.17, 1.21	<0.001
Other	0.73	0.71, 0.74	<0.001

^1^ OR—Odds Ratio, CI—Confidence Interval. ^2^ Reference group—White.

**Table 4:** Logistic Regression for SARS-CoV-2 Infection with apoA1 as Key Variable (*n* = 3589).

Variable	OR ^1^	95% CI ^1^	*p*-Value
ApoA1 value (mg/dL)	0.99	0.99, 1.00	<0.001
ESRD	1.74	0.94, 3.14	0.068
Obesity	0.58	0.48, 0.71	<0.001
Pregnancy	1.00	0.61, 1.60	0.995
Transplant	1.10	0.49, 2.28	0.815
Smoker	0.32	0.23, 0.44	<0.001
Cerebrovascular disease	0.90	0.62, 1.27	0.550
Chronic lung disease	0.76	0.61, 0.94	0.011
Congestive heart failure	0.89	0.54, 1.50	0.664
Dementia	1.17	0.65, 2.00	0.578
Depression	0.84	0.69, 1.03	0.088
Diabetes complicated	0.90	0.65, 1.25	0.531
Diabetes not complicated	1.57	1.19, 2.06	0.001
Heart Failure	1.14	0.72, 1.75	0.567
Hemiplegia	1.15	0.56, 2.23	0.683
HIV	0.28	0.13, 0.51	<0.001
Hypertension	1.25	1.01, 1.56	0.042
Cholesterol drug	0.20	0.12, 0.32	<0.001
Diabetes drug	2.55	1.70, 3.79	<0.001
Age	0.99	0.98, 1.00	0.044
Female	1.39	1.15, 1.68	<0.001
Race ^2^			
Black	0.95	0.72, 1.23	0.683
Asian	0.24	0.10, 0.46	<0.001
Hispanic	0.92	0.61, 1.36	0.673
Other	0.59	0.42, 0.81	0.002

^1^ OR—Odds Ratio, CI—Confidence Interval. ^2^ Reference group—White.

**Table 5:** Descriptive Analysis: COVID-19 Severity with HDL values.

Variable	Mild (*n* = 179,563)	Severe (*n* = 80,342)	*p*-Value ^1^
HDL value (mg/dL), Mean (SD)	51.72 (15.32)	48.46 (15.03)	<0.001
Age, Mean (SD)	51.27 (15.79)	61.01 (17.12)	<0.001
Female, *n* (%)	104,656 (58.28)	42,664 (53.10)	<0.001
Race/Ethnicity, *n* (%)			<0.001
White	134,254 (74.77)	50,333 (62.65)	
Asian	3116 (1.74)	1492 (1.86)	
Black	18,944 (10.55)	15,322 (19.07)	
Hispanic	10,497 (5.85)	6548 (8.15)	
Other	12,752 (7.10)	6647 (8.27)	
Pregnancy (yes/no), *n* (%)	7442 (4.14)	2705 (3.37)	<0.001
Smoking (yes/no), *n* (%)	4773 (2.66)	4319 (5.38)	<0.001
Comorbidity (yes/no), *n* (%)			
Obesity	76,600 (42.66)	42,594 (53.02)	<0.001
Hypertension	75,357 (41.97)	54,318 (67.61)	<0.001
Diabetes complicated	17,831 (9.93)	21,916 (27.28)	<0.001
Diabetes not complicated	36,325 (20.23)	32,488 (40.44)	<0.001
Cerebrovascular disease	7041 (3.92)	11,031 (13.73)	<0.001
Chronic lung disease	25,909 (14.43)	26,604 (33.11)	<0.001
Congestive heart failure	5823 (3.24)	14,787 (18.41)	<0.001
Heart Failure	9377 (5.22)	19,236 (23.94)	<0.001
Hemiplegia	1417 (0.79)	3563 (4.43)	<0.001
HIV	1080 (0.60)	819 (1.02)	<0.001
Dementia	3384 (1.88)	6880 (8.56)	<0.001
Depression	32,663 (18.19)	21,580 (26.86)	<0.001
ESRD	696 (0.39)	3009 (3.75)	<0.001
Transplant	370 (0.21)	1129 (1.41)	<0.001
Cholesterol drug (yes/no), *n* (%)	5869 (3.27)	7084 (8.82)	<0.001
Diabetes drug (yes/no), *n* (%)	7588 (4.23)	7090 (8.82)	<0.001

^1^ Statistical tests performed: *t*-, Wilcoxon rank-sum, chi-squared or Fisher’s exact test of independence.

**Table 6:** Descriptive Analysis: COVID-19 Severity with apoA1 values.

Variable	Mild (*n* = 392)	Severe (*n* = 249)	*p*-Value ^1^
ApoA1 value (mg/dL), Mean (SD)	141.97 (29.24)	135.45 (33.30)	0.006
Age, Mean (SD)	51.45 (13.25)	55.42 (14.10)	<0.001
Female, *n* (%)	214 (54.59)	117 (46.99)	0.060
Race/Ethnicity, *n* (%)			0.002
White	300 (76.53)	157 (63.05)	
Asian	<20 (<5.10)	<20 (<8.03)	
Black	43 (10.97)	50 (20.08)	
Hispanic	<20 (<5.10)	<20 (<8.03)	
Other	27 (6.89)	21 (8.43)	
Pregnancy (yes/no), *n* (%)	20 (5.10)	<20 (<8.03)	0.159
Smoking (yes/no), *n* (%)	27 (6.89)	24 (9.64)	0.210
Comorbidity (yes/no), *n* (%)			
Obesity	160 (40.82)	124 (49.80)	0.026
Hypertension	204 (52.04)	173 (69.48)	<0.001
Diabetes complicated	42 (10.71)	92 (36.95)	<0.001
Diabetes not complicated	104 (26.53)	124 (49.80)	<0.001
Cerebrovascular disease	<20 (<5.10)	36 (14.46)	<0.001
Chronic lung disease	73 (18.62)	99 (39.76)	<0.001
Congestive heart failure	<20 (<5.10)	48 (19.28)	<0.001
Heart Failure	33 (8.42)	63 (25.30)	<0.001
Hemiplegia	<20 (<5.10)	<20 (<8.03)	<0.001
HIV	<20 (<5.10)	<20 (<8.03)	0.522
Dementia	<20 (<5.10)	<20 (<8.03)	0.140
Depression	111 (28.32)	102 (40.96)	<0.001
ESRD	<20 (<5.10)	<20 (<8.03)	<0.001
Transplant	0 (0.00)	<20 (<8.03)	<0.001
Cholesterol drug (yes/no), *n* (%)	<20 (<5.10)	<20 (<8.03)	0.001
Diabetes drug (yes/no), *n* (%)	25 (6.38)	30 (12.05)	0.012

^1^ Statistical tests performed: *t*-, Wilcoxon rank-sum, chi-squared or Fisher’s exact test of independence.

**Table 7:** Logistic Regression for COVID-19 Severity with HDL as Key Variable (*n* = 259,905).

Variable	OR ^1^	95% CI ^1^	*p*-Value
HDL value (mg/dL)	0.99	0.99, 0.99	<0.001
ESRD	3.13	2.85, 3.45	<0.001
Obesity	1.09	1.07, 1.12	<0.001
Pregnancy	1.76	1.68, 1.85	<0.001
Transplant	2.18	1.91, 2.51	<0.001
Smoker	1.33	1.27, 1.40	<0.001
Cerebrovascular disease	1.36	1.31, 1.41	<0.001
Chronic lung disease	1.70	1.67, 1.74	<0.001
Congestive heart failure	1.38	1.30, 1.46	<0.001
Dementia	1.79	1.70, 1.87	<0.001
Depression	1.28	1.25, 1.31	<0.001
Diabetes complicated	1.25	1.21, 1.29	<0.001
Diabetes not complicated	1.19	1.15, 1.22	<0.001
Heart Failure	1.66	1.58, 1.74	<0.001
Hemiplegia	1.78	1.65, 1.91	<0.001
HIV	1.32	1.20, 1.47	<0.001
Hypertension	1.15	1.13, 1.18	<0.001
Cholesterol drug	1.31	1.25, 1.36	<0.001
Diabetes drug	1.12	1.08, 1.17	<0.001
Age	1.03	1.03, 1.03	<0.001
Female	0.88	0.87, 0.90	<0.001
Race ^2^			
Black	2.02	1.96, 2.07	<0.001
Asian	1.52	1.42, 1.62	<0.001
Hispanic	1.86	1.80, 1.93	<0.001
Other	1.54	1.49, 1.59	<0.001

^1^ OR—Odds Ratio, CI—Confidence Interval. ^2^ Reference group—White.

**Table 8:** Logistic Regression for COVID-19 Severity with apoA1 as Key Variable (*n* = 641).

Variable	OR ^1^	95% CI ^1^	*p*-Value
ApoA1 value (mg/dL)	0.99	0.99, 1.00	0.075
ESRD	3.74	0.91, 20.2	0.087
Obesity	0.96	0.65, 1.42	0.841
Pregnancy	0.88	0.30, 2.33	0.798
Transplant ^2^	NA	NA, NA	NA
Smoker	0.75	0.36, 1.52	0.429
Cerebrovascular disease	2.12	1.03, 4.49	0.045
Chronic lung disease	2.08	1.33, 3.24	0.001
Congestive heart failure	2.41	0.83, 7.25	0.108
Dementia	0.79	0.25, 2.49	0.690
Depression	1.40	0.91, 2.13	0.122
Diabetes complicated	2.40	1.27, 4.56	0.007
Diabetes not complicated	1.06	0.62, 1.80	0.828
Heart Failure	0.73	0.27, 1.83	0.505
Hemiplegia	9.34	1.39, 190	0.051
HIV	0.77	0.16, 3.32	0.726
Hypertension	0.91	0.58, 1.40	0.660
Cholesterol drug	2.37	0.93, 6.44	0.076
Diabetes drug	0.85	0.41, 1.73	0.649
Age	1.00	0.9967, 0.9974	0.588
Female	0.81	0.54, 1.21	0.298
Race ^3^			
Black	1.74	1.02, 2.95	0.041
Asian	0.76	0.10, 3.62	0.746
Hispanic	1.98	0.91, 4.30	0.082
Other	1.38	0.68, 2.74	0.359

^1^ OR—Odds Ratio, CI—Confidence Interval. ^2^ No transplant subjects were observed in the mild group. ^3^ Reference group—White.

**Table 9:** Descriptive Analysis: Development of AKI with HDL values.

Variable	No (*n* = 241,214)	Yes (*n* = 18,691)	*p*-Value ^1^
HDL value (mg/dL), Mean (SD)	51.05 (15.29)	46.30 (14.83)	<0.001
Age, Mean (SD)	53.34 (16.61)	66.48 (14.61)	<0.001
Female, *n* (%)	138,933 (57.60)	8387 (44.87)	<0.001
Race/Ethnicity, *n* (%)			<0.001
White	173,427 (71.90)	11,160 (59.71)	
Asian	4287 (1.78)	321 (1.72)	
Black	29,461 (12.21)	4805 (25.71)	
Hispanic	15,734 (6.52)	1311 (7.01)	
Other	18,305 (7.59)	1094 (5.85)	
Pregnancy (yes/no), *n* (%)	9890 (4.10)	257 (1.37)	<0.001
Smoking (yes/no), *n* (%)	7576 (3.14)	1516 (8.11)	<0.001
Comorbidity (yes/no), *n* (%)			
Obesity	107,184 (44.44)	12,010 (64.26)	<0.001
Hypertension	112,498 (46.64)	17,177 (91.90)	<0.001
Diabetes complicated	30,180 (12.51)	9567 (51.19)	<0.001
Diabetes not complicated	57,358 (23.78)	11,455 (61.29)	<0.001
Cerebrovascular disease	13,561 (5.62)	4511 (24.13)	<0.001
Chronic lung disease	43,165 (17.89)	9348 (50.01)	<0.001
Congestive heart failure	13,024 (5.40)	7586 (40.59)	<0.001
Heart Failure	19,660 (8.15)	8953 (47.90)	<0.001
Hemiplegia	3358 (1.39)	1622 (8.68)	<0.001
HIV	1655 (0.69)	244 (1.31)	<0.001
Dementia	7530 (3.12)	2734 (14.63)	<0.001
Depression	47,607 (19.74)	6636 (35.50)	<0.001
Transplant	741 (0.31)	758 (4.06)	<0.001
Cholesterol drug (yes/no), *n* (%)	10,664 (4.42)	2289 (12.25)	<0.001
Diabetes drug (yes/no), *n* (%)	12,419 (5.15)	2259 (12.09)	<0.001

^1^ Statistical tests performed: *t*-, Wilcoxon rank-sum, chi-squared or Fisher’s exact test of independence.

**Table 10:** Descriptive Analysis: Development of AKI with apoA1 values.

Variable	No (*n* = 577)	Yes (*n* = 64)	*p*-Value ^1^
apoA1 value (mg/dL), Mean (SD)	139.96 (30.67)	134.72 (33.90)	0.085
Age, Mean (SD)	52.46 (13.94)	57.81 (10.41)	0.005
Female, *n* (%)	305 (52.86)	26 (40.62)	0.063
Race/Ethnicity, *n* (%)			0.294
White	417 (72.27)	40 (62.50)	
Black	81 (14.04)	<20 (<31.25)	
Hispanic	29 (5.03)	<20 (<31.25)	
Asian/Other ^2^	50 (8.67)	<20 (<31.25)	
Pregnancy (yes/no), *n* (%)	27 (4.68)	0 (0.00)	0.098
Smoking (yes/no), *n* (%)	38 (6.59)	<20 (<31.25)	<0.001
Comorbidity (yes/no), *n* (%)			
Obesity	244 (42.29)	40 (62.50)	0.002
Hypertension	319 (55.29)	58 (90.62)	<0.001
Diabetes complicated	100 (17.33)	34 (53.12)	<0.001
Diabetes not complicated	187 (32.41)	41 (64.06)	<0.001
Cerebrovascular disease	35 (6.07)	<20 (<31.25)	<0.001
Chronic lung disease	138 (23.92)	34 (53.12)	<0.001
Congestive heart failure	48 (8.32)	<20 (<31.25)	<0.001
Heart Failure	70 (12.13)	26 (40.62)	<0.001
Hemiplegia	<20 (<3.47)	<20 (<31.25)	<0.001
HIV	<20 (<3.47)	<20 (<31.25)	0.263
Dementia	<20 (<3.47)	<20 (<31.25)	0.026
Depression	179 (31.02)	34 (53.12)	<0.001
Transplant	<20 (<3.47)	<20 (<31.25)	<0.001
Cholesterol drug (yes/no), *n* (%)	22 (3.81)	<20 (<31.25)	0.317
Diabetes drug (yes/no), *n* (%)	47 (8.15)	<20 (<31.25)	0.238

^1^ Statistical tests performed: *t*-, Wilcoxon rank-sum, chi-squared or Fisher’s exact test of independence. ^2^ According to the N3C DUA, Asian (*n* < 20; <3.47%) was combined with Other to increase the cell size and avoid back-calculation. The statistical test was performed on the data before combining the two categories.

**Table 11:** Logistic Regression for Development of AKI with HDL as Key Variable (*n* = 259,905).

Variable	OR ^1^	95% CI ^1^	*p*-Value
HDL value (mg/dL)	0.99	0.99, 1.0	<0.001
Obesity	1.26	1.21, 1.31	<0.001
Pregnancy	0.99	0.85, 1.14	0.859
Transplant	6.58	5.84, 7.43	<0.001
Smoker	1.36	1.27, 1.46	<0.001
Cerebrovascular disease	1.33	1.27, 1.40	<0.001
Chronic lung disease	1.73	1.66, 1.79	<0.001
Congestive heart failure	1.83	1.70, 1.96	<0.001
Dementia	1.54	1.45, 1.63	<0.001
Depression	1.41	1.35, 1.46	<0.001
Diabetes complicated	2.13	2.02, 2.24	<0.001
Diabetes not complicated	1.14	1.08, 1.20	<0.001
Heart Failure	1.68	1.57, 1.80	<0.001
Hemiplegia	1.47	1.36, 1.59	<0.001
HIV	1.36	1.15, 1.59	<0.001
Hypertension	3.35	3.16, 3.56	<0.001
Cholesterol drug	1.02	0.96, 1.08	0.462
Diabetes drug	0.92	0.87, 0.98	0.005
Age	1.03	1.03, 1.03	<0.001
Female	0.64	0.62, 0.66	<0.001
Race ^2^			
Black	2.05	1.96, 2.14	<0.001
Asian	1.43	1.25, 1.62	<0.001
Hispanic	1.34	1.25, 1.44	<0.001
Other	0.99	0.92, 1.06	0.812

^1^ OR—Odds Ratio, CI—Confidence Interval. ^2^ Reference group—White.

**Table 12:** Logistic Regression for Development of AKI with apoA1 as Key Variable (*n* = 641).

Variable	OR ^1^	95% CI ^1^	*p*-Value
ApoA1 value (mg/dL)	1.00	0.99, 1.01	0.872
Obesity	1.46	0.75, 2.87	0.271
Pregnancy ^2^	NA	NA, NA	NA
Transplant	15.00	3.82, 70.1	<0.001
Smoker	2.31	0.91, 5.60	0.068
Cerebrovascular disease	2.35	0.98, 5.39	0.049
Chronic lung disease	1.75	0.89, 3.42	0.100
Congestive heart failure	0.75	0.23, 2.67	0.647
Dementia	1.38	0.30, 5.31	0.655
Depression	1.49	0.75, 2.93	0.250
Diabetes complicated	1.77	0.68, 4.84	0.250
Diabetes not complicated	0.82	0.30, 2.04	0.683
Heart Failure	2.30	0.71, 6.62	0.139
Hemiplegia	3.38	0.81, 14.3	0.091
HIV	0.64	0.07, 4.25	0.667
Hypertension	3.26	1.28, 9.54	0.019
Cholesterol drug	0.89	0.19, 3.20	0.874
Diabetes drug	0.60	0.21, 1.59	0.325
Age	1.00	0.97, 1.03	0.991
Female	0.70	0.36, 1.37	0.305
Race ^3^			
Black	0.96	0.40, 2.13	0.925
Asian ^2^	NA	NA, NA	NA
Hispanic	3.13	0.94, 9.15	0.046
Other	1.26	0.40, 3.47	0.667

^1^ OR—Odds Ratio, CI—Confidence Interval. ^2^ No pregnancy and Asian subjects were observed in the AKI group. ^3^ Reference group—White.

## Data Availability

The National Institute of Health’s National COVID Cohort Collaborative (N3C) data used in this study is available upon application at https://covid.cd2h.org/(accessed on 11 November 2022).

## Appendix A

**Table A1:** Variables used in the analysis.

Variable	Measure	Value
HDL	OMOP Concept ID	3007070
APOA1	OMOP Concept ID	37024265
Acute Kidney Injury	ICD-10	N17.0, N17.1, N17.2, N17.8, N17.9
Pregnancy	OMOP Concept ID	4218813, 4239938, 4244438
Smoking	OMOP Concept ID	4298794, 44789712, 4269997, 4190573, 4046886, 40486696, 4216174, 4052948, 40486518
Obesity	OMOP Concept ID	4215968, 438731, 4216214, 437525
Hypertension	OMOP Concept ID	316866, 4071202, 42709887
Diabetes Complicated	OMOP Concept ID	Include: 442793, 44793113, 4096041, 45772060, 4321756, 36715417, 45769832, 43531616, 4096671, 4096670, 37204818, 40482883, 4265913 Exclude: 4034964, 42536605, 43531597, 37016350, 45769875, 42538715, 4082347, 37311673, 45757363, 4033942, 44789319
Diabetes not Complicated	OMOP Concept ID	Include: 201820 Exclude: 192279, 43531616, 44793113, 442793, 44805490, 44812346, 36715417, 42535539, 4099651
Cerebrovascular Disease	OMOP Concept ID	381591, 434056
Chronic Lung Disease	OMOP Concept ID	Include: 4027836, 4050877, 4121621, 4120270, 37116690, 37309675, 317009, 4283942, 42537657, 45763749, 257775, 4230358, 4148529, 45767051, 258780, 261889, 37311779, 255059, 4233477, 4052549, 42536541, 40482019, 437313, 4322024, 255841, 45768983, 40480461, 438791, 44782927, 4204998, 4232302, 45768892, 40483342, 37311903, 441267, 45769020, 4103099, 4105601, 256449, 762964, 4197819, 45769386, 45768915, 4341520, 3655634, 4052553, 4071743, 438782, 4138307, 4273378, 37116655, 4172303, 42537658, 4112813, 4306635, 4174275, 4084955, 4232485, 765431, 45769019, 36674196, 44810118, 45772934, 4141669, 4203619, 255573, 4137505, 4102140, 4144583, 45768987, 4500876, 3655347, 45771017, 4270139, 4078695, 4110637, 45771019, 44805713, 4028118, 42539687, 45769146, 44802278, 4119786, 4116317, 4052550, 36715501, 3655969, 4186898, 46272927, 4140605, 46273640, 45768996, 4052548, 4173466, 4173466, 4050874, 4309350, 46270493 Exclude: 4198434, 43020840, 44782989, 4337510
Congestive Heart Failure	OMOP Concept ID	319835
Heart Failure	OMOP Concept ID	Include: 316139, 4236658, 321319 Exclude: 43020893
Hemiplegia	OMOP Concept ID	43531638, 43531639
HIV	OMOP Concept ID	439727
Dementia	OMOP Concept ID	4182210, 4236296, 4250118, 4233045, 4236297
Depression	OMOP Concept ID	Include: 4327217, 440383, 4298317 Exclude: 4224940, 436665
ESRD	OMOP Concept ID	193782, 45769906, 4782717, 46273164, 4030520, 4128200, 37018886, 43020455, 43021864, 45769904, 762973
Transplant	OMOP Concept ID	Include: 42538119, 42898004, 2741982, 42897987, 42897992, 2727298, 42898011, 42538117, 4287985, 42539502, 4121274, 2741697, 2741958, 42898007, 42537745, 2741961, 42897991, 42898012, 2741964, 2727190, 1524123, 2741979, 42897988, 42898008, 42898009, 4127554, 42898006, 2741970, 2750767, 1524124, 4341658, 42897993, 4208341, 42898003, 42538118, 2752914, 1524116, 44791468, 42898005, 1524118, 42897986, 2741973, 4121617, 1524122, 1524117, 2741976, 2774520, 42897990, 42539698, 42537742, 2774519, 42897989, 2774517, 2750764, 2774522 Exclude: 4265621, 44810212
Cholesterol Drug	Drug Name	Niacin/nicotinic acid (Niacor, Nicobid, Nicolar, Niaspan), Gemfibrozil (Lopid), Fenofibrate (Tricor), Clofibrate (Atromid-S), Atorvastatin (Lipitor), Simvastatin (Zocor), Prevastatin (Pravachol), Lovastatin (Mevacor), Fluvastatin (Lescol), Rosuvastatin (Crestor), Pitavastatin (Livalo)
Diabetes Drug	Drug Name	Repaglinide (Prandin), Nateglinide (Starlix), Glipizide (Glucotrol XL), Glimepiride (Amaryl), Glyburide (DiaBeta, Glynase), Saxagliptin (Onglyza), Sitagliptin (Januvia), Linagliptin (Tradjenta), Alogliptin (Nesina), Metformin (Fortamet, Glumetza, others), Rosiglitazone (Avandia), Pioglitazone (Actos), Acarbose (Precose), Miglitol (Glyset), Canagliflozin (Invokana), Dapagliflozin (Farxiga), Empagliflozin (Jardiance), Ertugliflozin (Steglatro), Colesevelam (Welchol), Pramlintide (Symlin), Dulaglutide (Trulicity), Exenatide (Byetta, Bydureon Bcise), Liraglutide (Saxenda, Victoza), Lixisenatide (Adlyxin), Semaglutide (Ozempic, Rybelsus, Wegovy)

Note: The definitions of the Observational Medical Outcomes Partnership (OMOP) concept IDs can be found via https://athena.ohdsi.org/search-terms/terms (accessed on 11 November 2022).

## Appendix B

1. Disclaimer

The N3C Publication Committee confirmed that this manuscript <msid: 1036.53> is in accordance with N3C data use and attribution policies; however, this content is solely the responsibility of the authors and does not necessarily represent the official views of the National Institutes of Health or the N3C program.

2. IRB

The N3C data transfer to NCATS was performed under a Johns Hopkins University Reliance Protocol # IRB00249128 or individual site agreements with NIH. The N3C Data Enclave is managed under the authority of the NIH; information can be found at https://ncats.nih.gov/n3c/resources.

3. Individual Acknowledgements for Core Contributors

We gratefully acknowledge the following core contributors to N3C:

Adam B. Wilcox, Adam M. Lee, Alexis Graves, Alfred (Jerrod) Anzalone, Amin Manna, Amit Saha, Amy Olex, Andrea Zhou, Andrew E. Williams, Andrew Southerland, Andrew T. Girvin, Anita Walden, Anjali A. Sharathkumar, Benjamin Amor, Benjamin Bates, Brian Hendricks, Brijesh Patel, Caleb Alexander, Carolyn Bramante, Cavin Ward-Caviness, Charisse Madlock-Brown, Christine Suver, Christopher Chute, Christopher Dillon, Chunlei Wu, Clare Schmitt, Cliff Takemoto, Dan Housman, Davera Gabriel, David A. Eichmann, Diego Mazzotti, Don Brown, Eilis Boudreau, Elaine Hill, Elizabeth Zampino, Emily Carlson Marti, Emily R. Pfaff, Evan French, Farrukh M Koraishy, Federico Mariona, Fred Prior, George Sokos, Greg Martin, Harold Lehmann, Heidi Spratt, Hemalkumar Mehta, Hongfang Liu, Hythem Sidky, J.W. Awori Hayanga, Jami Pincavitch, Jaylyn Clark, Jeremy Richard Harper, Jessica Islam, Jin Ge, Joel Gagnier, Joel H. Saltz, Joel Saltz, Johanna Loomba, John Buse, Jomol Mathew, Joni L. Rutter, Julie A. McMurry, Justin Guinney, Justin Starren, Karen Crowley, Katie Rebecca Bradwell, Kellie M. Walters, Ken Wilkins, Kenneth R. Gersing, Kenrick Dwain Cato, Kimberly Murray, Kristin Kostka, Lavance Northington, Lee Allan Pyles, Leonie Misquitta, Lesley Cottrell, Lili Portilla, Mariam Deacy, Mark M. Bissell, Marshall Clark, Mary Emmett, Mary Morrison Saltz, Matvey B. Palchuk, Melissa A. Haendel, Meredith Adams, Meredith Temple-O’Connor, Michael G. Kurilla, Michele Morris, Nabeel Qureshi, Nasia Safdar, Nicole Garbarini, Noha Sharafeldin, Ofer Sadan, Patricia A. Francis, Penny Wung Burgoon, Peter Robinson, Philip R.O. Payne, Rafael Fuentes, Randeep Jawa, Rebecca Erwin-Cohen, Rena Patel, Richard A. Moffitt, Richard L. Zhu, Rishi Kamaleswaran, Robert Hurley, Robert T. Miller, Saiju Pyarajan, Sam G. Michael, Samuel Bozzette, Sandeep Mallipattu, Satyanarayana Vedula, Scott Chapman, Shawn T. O’Neil, Soko Setoguchi, Stephanie S. Hong, Steve Johnson, Tellen D. Bennett, Tiffany Callahan, Umit Topaloglu, Usman Sheikh, Valery Gordon, Vignesh Subbian, Warren A. Kibbe, Wenndy Hernandez, Will Beasley, Will Cooper, William Hillegass, Xiaohan Tanner Zhang. Details of contributions available at covid.cd2h.org/core-contributors

4. Data Partners with Released Data

The following institutions whose data is released or pending:

Available: Advocate Health Care Network—UL1TR002389: The Institute for Translational Medicine (ITM) • Boston University Medical Campus—UL1TR001430: Boston University Clinical and Translational Science Institute • Brown University—U54GM115677: Advance Clinical Translational Research (Advance-CTR) • Carilion Clinic—UL1TR003015: iTHRIV Integrated Translational health Research Institute of Virginia • Charleston Area Medical Center—U54GM104942: West Virginia Clinical and Translational Science Institute (WVCTSI) • Children’s Hospital Colorado—UL1TR002535: Colorado Clinical and Translational Sciences Institute • Columbia University Irving Medical Center—UL1TR001873: Irving Institute for Clinical and Translational Research • Duke University—UL1TR002553: Duke Clinical and Translational Science Institute • George Washington Children’s Research Institute—UL1TR001876: Clinical and Translational Science Institute at Children’s National (CTSA-CN) • George Washington University—UL1TR001876: Clinical and Translational Science Institute at Children’s National (CTSA-CN) • Indiana University School of Medicine—UL1TR002529: Indiana Clinical and Translational Science Institute • Johns Hopkins University—UL1TR003098: Johns Hopkins Institute for Clinical and Translational Research • Loyola Medicine—Loyola University Medical Center • Loyola University Medical Center—UL1TR002389: The Institute for Translational Medicine (ITM) • Maine Medical Center—U54GM115516: Northern New England Clinical & Translational Research (NNE-CTR) Network • Massachusetts General Brigham—UL1TR002541: Harvard Catalyst • Mayo Clinic Rochester—UL1TR002377: Mayo Clinic Center for Clinical and Translational Science (CCaTS) • Medical University of South Carolina—UL1TR001450: South Carolina Clinical & Translational Research Institute (SCTR) • Montefiore Medical Center—UL1TR002556: Institute for Clinical and Translational Research at Einstein and Montefiore • Nemours—U54GM104941: Delaware CTR ACCEL Program • NorthShore University HealthSystem—UL1TR002389: The Institute for Translational Medicine (ITM) • Northwestern University at Chicago—UL1TR001422: Northwestern University Clinical and Translational Science Institute (NUCATS) • OCHIN—INV-018455: Bill and Melinda Gates Foundation grant to Sage Bionetworks • Oregon Health & Science University—UL1TR002369: Oregon Clinical and Translational Research Institute • Penn State Health Milton S. Hershey Medical Center—UL1TR002014: Penn State Clinical and Translational Science Institute • Rush University Medical Center—UL1TR002389: The Institute for Translational Medicine (ITM) • Rutgers, The State University of New Jersey—UL1TR003017: New Jersey Alliance for Clinical and Translational Science • Stony Brook University—U24TR002306 • The Ohio State University—UL1TR002733: Center for Clinical and Translational Science • The State University of New York at Buffalo—UL1TR001412: Clinical and Translational Science Institute • The University of Chicago—UL1TR002389: The Institute for Translational Medicine (ITM) • The University of Iowa—UL1TR002537: Institute for Clinical and Translational Science • The University of Miami Leonard M. Miller School of Medicine—UL1TR002736: University of Miami Clinical and Translational Science Institute • The University of Michigan at Ann Arbor—UL1TR002240: Michigan Institute for Clinical and Health Research • The University of Texas Health Science Center at Houston—UL1TR003167: Center for Clinical and Translational Sciences (CCTS) • The University of Texas Medical Branch at Galveston—UL1TR001439: The Institute for Translational Sciences • The University of Utah—UL1TR002538: Uhealth Center for Clinical and Translational Science • Tufts Medical Center—UL1TR002544: Tufts Clinical and Translational Science Institute • Tulane University—UL1TR003096: Center for Clinical and Translational Science • University Medical Center New Orleans—U54GM104940: Louisiana Clinical and Translational Science (LA CaTS) Center • University of Alabama at Birmingham—UL1TR003096: Center for Clinical and Translational Science • University of Arkansas for Medical Sciences—UL1TR003107: UAMS Translational Research Institute • University of Cincinnati—UL1TR001425: Center for Clinical and Translational Science and Training • University of Colorado Denver, Anschutz Medical Campus—UL1TR002535: Colorado Clinical and Translational Sciences Institute • University of Illinois at Chicago—UL1TR002003: UIC Center for Clinical and Translational Science • University of Kansas Medical Center—UL1TR002366: Frontiers: University of Kansas Clinical and Translational Science Institute • University of Kentucky—UL1TR001998: UK Center for Clinical and Translational Science • University of Massachusetts Medical School Worcester—UL1TR001453: The UMass Center for Clinical and Translational Science (UMCCTS) • University of Minnesota—UL1TR002494: Clinical and Translational Science Institute • University of Mississippi Medical Center—U54GM115428: Mississippi Center for Clinical and Translational Research (CCTR) • University of Nebraska Medical Center—U54GM115458: Great Plains IDeA-Clinical & Translational Research • University of North Carolina at Chapel Hill—UL1TR002489: North Carolina Translational and Clinical Science Institute • University of Oklahoma Health Sciences Center—U54GM104938: Oklahoma Clinical and Translational Science Institute (OCTSI) • University of Rochester—UL1TR002001: UR Clinical & Translational Science Institute • University of Southern California—UL1TR001855: The Southern California Clinical and Translational Science Institute (SC CTSI) • University of Vermont—U54GM115516: Northern New England Clinical & Translational Research (NNE-CTR) Network • University of Virginia—UL1TR003015: iTHRIV Integrated Translational health Research Institute of Virginia • University of Washington—UL1TR002319: Institute of Translational Health Sciences • University of Wisconsin-Madison—UL1TR002373: UW Institute for Clinical and Translational Research • Vanderbilt University Medical Center—UL1TR002243: Vanderbilt Institute for Clinical and Translational Research • Virginia Commonwealth University—UL1TR002649: C. Kenneth and Dianne Wright Center for Clinical and Translational Research • Wake Forest University Health Sciences—UL1TR001420: Wake Forest Clinical and Translational Science Institute • Washington University in St. Louis—UL1TR002345: Institute of Clinical and Translational Sciences • Weill Medical College of Cornell University—UL1TR002384: Weill Cornell Medicine Clinical and Translational Science Center • West Virginia University—U54GM104942: West Virginia Clinical and Translational Science Institute (WVCTSI)

Submitted: Icahn School of Medicine at Mount Sinai—UL1TR001433: ConduITS Institute for Translational Sciences • The University of Texas Health Science Center at Tyler—UL1TR003167: Center for Clinical and Translational Sciences (CCTS) • University of California, Davis—UL1TR001860: UC Davis Health Clinical and Translational Science Center • University of California, Irvine—UL1TR001414: The UC Irvine Institute for Clinical and Translational Science (ICTS) • University of California, Los Angeles—UL1TR001881: UCLA Clinical Translational Science Institute • University of California, San Diego—UL1TR001442: Altman Clinical and Translational Research Institute • University of California, San Francisco—UL1TR001872: UCSF Clinical and Translational Science Institute

Pending: Arkansas Children’s Hospital—UL1TR003107: UAMS Translational Research Institute • Baylor College of Medicine—None (Voluntary) • Children’s Hospital of Philadelphia—UL1TR001878: Institute for Translational Medicine and Therapeutics • Cincinnati Children’s Hospital Medical Center—UL1TR001425: Center for Clinical and Translational Science and Training • Emory University—UL1TR002378: Georgia Clinical and Translational Science Alliance • HonorHealth—None (Voluntary) • Loyola University Chicago—UL1TR002389: The Institute for Translational Medicine (ITM) • Medical College of Wisconsin—UL1TR001436: Clinical and Translational Science Institute of Southeast Wisconsin • MedStar Health Research Institute—UL1TR001409: The Georgetown-Howard Universities Center for Clinical and Translational Science (GHUCCTS) • MetroHealth—None (Voluntary) • Montana State University—U54GM115371: American Indian/Alaska Native CTR • NYU Langone Medical Center—UL1TR001445: Langone Health’s Clinical and Translational Science Institute • Ochsner Medical Center—U54GM104940: Louisiana Clinical and Translational Science (LA CaTS) Center • Regenstrief Institute—UL1TR002529: Indiana Clinical and Translational Science Institute • Sanford Research—None (Voluntary) • Stanford University—UL1TR003142: Spectrum: The Stanford Center for Clinical and Translational Research and Education • The Rockefeller University—UL1TR001866: Center for Clinical and Translational Science • The Scripps Research Institute—UL1TR002550: Scripps Research Translational Institute • University of Florida—UL1TR001427: UF Clinical and Translational Science Institute • University of New Mexico Health Sciences Center—UL1TR001449: University of New Mexico Clinical and Translational Science Center • University of Texas Health Science Center at San Antonio—UL1TR002645: Institute for Integration of Medicine and Science • Yale New Haven Hospital—UL1TR001863: Yale Center for Clinical Investigation.
